# Cardiovascular Effects of Adding Adrenaline to Arthroscopic Knee Irrigation Fluid in Canine Diagnostic Procedures

**DOI:** 10.3390/ani15111544

**Published:** 2025-05-25

**Authors:** Ana Zapata, Claudio Iván Serra Aguado, José Ignacio Redondo, José Román Soto Muñoz, José Sansano-Maestre, Rocío Fernández-Parra

**Affiliations:** 1 Hospital Veterinario de Referencia UCV, Departamento de Medicina y Cirugía Animal, Facultad de Veterinaria y Ciencias Experimentales, Universidad Católica de Valencia San Vicente Mártir, 46018 Valencia, Spain; 2 Programa de Doctorado en Ciencias de la Vida y del Medio Natural, Escuela de Doctorado, Universidad Católica de Valencia San Vicente el Mártir, 46002 Valencia, Spain; 3 Departamento Medicina y Cirugía Animal, Facultad de Veterinaria y Ciencias Experimentales, Universidad Católica de Valencia San Vicente Mártir, 46002 Valencia, Spain; 4 Departamento de Medicina y Cirugía Animal, Facultad de Veterinaria, Universidad Cardenal Herrera-CEU, CEU Universities, C/Tirant lo Blanch, 7, Alfara del Patriarca, 46115 Valencia, Spain; 5 Veterinary Specialist Ireland, A83 EV27 Summerhill, Ireland; 6 Departamento de Producción Animal y Salud Pública. Universidad Católica de Valencia San Vicente Mártir, 46002 Valencia, Spain

**Keywords:** knee arthroscopy, dog, epinephrine, local hemostasia, intra-articular bleeding, safety, image quality, MostCare

## Abstract

Background: Adrenaline is used as a local haemostatic in human arthroscopy, due to its peripheral vasoconstrictive properties, which enhance image quality with minimal cardiovascular side effects. This study aims to evaluate the effects of adrenaline in arthroscopic irrigation fluid and asses its impact on image visibility. Methods: A prospective, double-blind, randomised clinical trial was conducted on 20 knees from 18 dogs undergoing diagnostic arthroscopies. Dogs were randomly assigned to two groups: group A (received lactate Ringer (LR) with adrenaline 0.33 mg L^−1^) and group C (received LR with 0.33 mL of saline solution). Cardiovascular parameters were recorded using a multiparametric monitor, with advanced hemodynamic parameters assessed using the PRAM method (MostCare^®^) in half of the dogs, and glucose, cortisol, and adrenaline levels were measured at various time points. Hemoglobin concentration was calculated based on the residual fluid and image quality was evaluated by a blinded surgeon using a visual analog scale (VAS). Results: No significant differences between groups in cardiovascular parameters, glucose, cortisol, adrenaline, hemoglobin, or VAS scores; only time differences for cortisol and adrenaline. Conclusions: The use of adrenaline at 0.33 mg L^−1^ in arthroscopic irrigation fluid does not cause significant cardiovascular changes or improve image quality, confirming it is safe at this concentration.

## Simple Summary

Intra-articular bleeding resulting from the creation of the arthroscopic portal and manipulation of joint tissues can significantly impair visual clarity during the procedure. In human medicine, adrenaline is commonly added to the irrigation fluid to enhance visibility. However, this practice carries a minimal risk of inducing hypertension and/or tachycardia (with or without arrhythmias). This study aims to assess whether the use of adrenaline at a concentration of 0.33 mg L^−1^ in the arthroscopic irrigation fluid would cause hemodynamic alterations in dogs. In half of the dogs, advanced hemodynamic parameters were monitored using the pressure recording analytical method (PRAM) with the MostCare^®^ system. No significant differences were observed in cardiovascular parameters between groups at different time points or within the same group over time. In conclusion, the use of adrenaline at 0.33 mg L^−1^ as a haemostatic agent in arthroscopic irrigation fluid does not produce significant cardiovascular alterations in dogs. Similarly, advanced monitoring appears to show consistent results, with no alterations in hemodynamic parameters.

## 1. Introduction

Arthroscopy is a minimally invasive procedure that facilitates the diagnosis and treatment of intra-articular pathologies [1]. This technique provides a magnified image to a high-definition monitor. At the same time, the joint is distended with liquid, allowing comprehensive visualization of the intra-articular structures, including soft tissues and articular cartilage, in their most natural environment [2,3,4]. In canines, this technique has grown exponentially in recent years, driven by owner demand and technological advances such as high-resolution cameras, fibre optics, and cold light sources [5]. Today, it is considered the gold standard technique for evaluating intra-articular structures [6,7].

Although arthroscopy is a procedure with a low complication rate [8], a recent study found that 17.1% of dogs are at risk of “minor or minimal” perioperative complications [9]. One such complication is intra-articular bleeding, which occurs after the creation of the arthroscopic portal and during the manipulation of joint tissues. This bleeding dilutes with the irrigation fluid, negatively affecting the quality of the arthroscopic image [2]. In human medicine and horses, intra-articular haemorrhage compromises joint visibility, prolongs surgery, and increases complexity, potentially leading to diagnostic errors and tissue damage. Additionally, it has been associated with a higher risk of postoperative complications, susceptibility to infection, articular cartilage toxicity, subsequent synovitis, and increased postoperative pain [10,11,12]. Consequently, methods have been developed to minimise the presence of blood in the joint, such as thermal coagulation (electrocautery), pump irrigation systems, tourniquets [13,14], application of locoregional anaesthesia techniques [15,16,17], and/or administration of a haemostatic drug. In human medicine, drugs such as adrenaline or noradrenaline are applied through arthroscopic irrigation fluid [18,19,20,21], and intravenous and/or intra-articular tranexamic acid is used at various stages of the perioperative period [22,23,24].

Adrenaline is an endogenous catecholamine produced physiologically by the adrenal glands [25]. It is a powerful inotrope, positive chronotrope, and vasopressor, whose application can lead to tachycardia and hypertension. Peripheral vasoconstriction is induced by specific vascular smooth muscle membrane receptors *α*_1_ and *α*_2_ stimulation [26]. In veterinary medicine, adrenaline is commonly administrated intravenously for the treatment of anaphylactic shock and cardiovascular resuscitation [27,28]. However, it is also used locally to act as a haemostatic agent to reduce bleeding through its tamponade effect and peripheral vasoconstriction of tissue [29,30,31]. In human medicine, the use of adrenaline during the arthroscopic procedure, due to haemostatic properties at concentrations of 0.33 mg L^−1^ in the irrigation fluid, is considered a common and safe technique during anaesthetic management. It has been associated with a lower degree of intra-articular bleeding. Evidence supports it leads to higher quality images, decreased operative time, reduced total volume of irrigation fluid, and improved post-surgical pain [17,19,20,32,33]. The risk of hypertension, tachycardia with or without ventricular arrhythmia, and/or pulmonary oedema are al minimal or almost non-existent [34,35]. In veterinary medicine, there are no publications on the use of these drugs and their benefits, although their use is recommended by some authors based on their clinical experience, as in the case of using ephedrine in arthroscopic irrigation fluid in horses [11].

Therefore, this study aims to evaluate the impact of adding adrenaline to the intra-articular lavage fluid on cardiorespiratory parameters during arthroscopic procedures, while also determine how the quality of the arthroscopic image can be influenced. Our hypothesis suggests that the use of adrenaline at a dose of 0.33 mg L^−1^ diluted in the arthroscopic irrigation fluid will decrease intra-articular bleeding with minimal involvement of the cardiovascular system and improve arthroscopy image quality.

## 2. Materials and Methods

A prospective, randomised, double-blinded study (the traumatologist and the statistician, but not the anaesthesiologist) was implemented to assess the impact of two different joint lavage protocols on dogs undergoing diagnostic arthroscopy in the period from January 2022 and March 2024. This exception for the anaesthesiologist was justified by the need for them to be aware of the drugs administered to the dogs to make informed decisions regarding potential complications. The present study was approved by the Committee for Animal Experimentation of the Universidad Católica de Valencia San Vicente Mártir (CEEAUCV2102/7 January 2022).

### 2.1. Case Selection

Twenty-two knees from 20 client-owner dogs at the UCV Veterinary Referral Hospital were enrolled in the study for diagnostic knee arthroscopy prior to TTA (tibial tuberosity advancement) or TPLO (tibial plateau levelling osteotomy). Inclusion criteria required signed informed consent from the owner, dog age between 6 months and 15 years, locoregional blockade and having an arterial catheter placed. Exclusion criteria were an improper handling of plasma samples for measurement of adrenaline and a classification of the American Society of Anaesthesiologists (ASA) ≥ III. Eligible dogs were considered healthy, with no diagnosed or suspected systemic disease, based on physical examination and bloodwork results.

### 2.2. Anaesthetic Protocol

Animals were fasted for 12 h preoperatively. All dogs were premedicated with medetomidine 0.01 mg kg^−1^ (Domtor 1 mg mL^−1^, Ecuphar Veterinaria S.L.U., Barcelona, Spain) and methadone 0.2 mg kg^−1^ (Metasedin 10 mg mL^−1^, Esteve Pharmaceuticals, S.A., Barcelona, Spain) mixed in the same syringe intramuscularly (IM). Twenty minutes after premedication, animals were preoxygenated via face mask and a catheter (Introcan Certo, B.Braun VetCare SA, Barcelona, Spain) was placed in the left or right cephalic vein. A blood sample was obtained from the catheter and added to a citrate tube to measure clotting times. A lactated Ringer’s infusion (LR) (Lactato-RingerVet, B.Braun VetCare SA, Barcelona, Spain) was started at a constant rate of 5 mL kg^−1^ h^−1^. The dogs were induced with ketamine 1 mg kg^−1^ (Imalgene 100 mg/mL, Boehringer Ingelheim Animal Health S.A.U, Barcelona, Spain) and propofol to dose-effect intravenously (IV) (Propovet multidose 10 mg mL^−1^, Ecuphar Veterinari S.L.U., Barcelona, Spain) to obtain orotracheal intubation. Anaesthesia was maintained with isoflurane (Isovet 1000 mg g^−1^, Piramal Critical Care B.V., Voorschoten, Netherlands) vaporised in an air and oxygen admixture with an oxygen-inspired fraction (FiO_2_) > 0.4. The maintenance with isoflurane during all the procedures was according to the dog’s requirements. Volume-controlled mechanical ventilation was initiated in dogs deemed necessary, including obese dogs, those experiencing apnoea after induction, or dogs with moderate hypercapnia (PCO_2_ > 55 mmHg). Prophilactic Cefazoline 22 mg kg^−1^ (Cefazolina Normon, Laboratorios Normon S.A., Madrid, Spain) was administrated IV after induction. All dogs were positioned in lateral recumbency with the operative side uppermost. The extremity was clipped, and the skin was disinfected. An ultrasound-guided nerve block was performed on the sciatic and saphenous nerves, as described by Portela et al. (2018) [36] using 0.15 mL kg^−1^ per injection point of 0.25% bupivacaine (Bupivacaine 5 mg mL^−1^, B.Braun VetCare SA, Barcelona, Spain), administered with a nerve stimulator needle (Stimuplex Ultra 360, B. Braun Melsungen AG, Melsungen, Germany). A catheter was placed in the metatarsal artery of the non-operated hind limb to measure invasive blood pressure.

### 2.3. Arthroscopic Procedure and Recorded Parameters

The arthroscopic procedure was performed using the EndoVue^®^ medical monitor (NDS Surgical Imaging, Zevenhuizen, The Netherlands), a modular camera system with a connection module (Karl Storz-Endoscope Image1 S™ Connect TC 200, Tuttlingen, Germany) and a head link module (Image1 S™H3-Link TC300, Tuttlingen, Germany), a cold light source (Karl Storz-Endoscope SCE D-light P 201337 20, Tuttlingen, Germany), a camera head with 3 chips (Image HD Karl Storz-Endoscope, Tuttlingen, Germany) and different diameters of 2.4 mm or 2.7 mm optics with 30 degrees lens angle. A continuous fluid irrigation (LR) system with an SCE arthropump 283300 20 roller pump (Karl Storz-Endoscope, Tuttlingen, Germany) was used with initial pressure parameters of 60 mmHg and a speed of 300 mL min^−1^. The pressure and flow rate parameters were adjusted as needed during the arthroscopy to further distend the joint or improve visualization.

Dogs were randomly allocated to one of two groups, using a computer-generated randomization list (https://pinetools.com/es/aleatorizar-lista, accessed on 7 January 2022): a control group (group C) and an adrenaline group (group A). In group A (adrenaline 0.33 mg L^−1^), the arthroscopy was performed with 0.33 mL of adrenaline (Adrenalina 1 mg mL^−1^, B. Braun VetCare SA., Spain) diluted in one litre of LR of irrigation fluid as described in the investigation of Montfoort et al. (2016) [17]. In the control group, the arthroscopy was performed using one litre of LR with 0.33 mL of physiological saline solution. Two minutes after starting the arthroscopy, the irrigation fluid (LR) was changed for the study irrigation fluid. During the study, the following parameters were evaluated: speed and pressure of the pumping system, heart rate (HR), respiratory rate (RR), systolic arterial pressure (SAP), mean arterial pressure (MAP), diastolic arterial pressure (DAP) (General Electric B40 V2, General Electric HealthCare, S.A., Madrid, Spain), cardiac output (CO), stroke volume (SV), systemic vascular resistance (SVR), maximum pressure variation over time (dP/dtmax), systolic-pressure variation (SPV), pulse-pressure variation (PPV), and cardiac cycle efficiency (CCE) using the pressure recording analytical method (PRAM) (MostCare^®^, Vygon España, Valencia, Spain). The recording of the parameters was carried out at T_0_ (2 min before starting the arthroscopy), T_2_ (within 2 min of waiting after the entrance of the optic), T_5_ (after 5 min of starting with the study irrigation fluid), and then every 5 min until the end of the procedure (T_10_, T_15_, T_20_ and T_25_). During the arthroscopic procedure, if the dog presented nociception (an increase of >20% in cardiorespiratory values for more than 1 min) it was treated with fentanyl boluses 1–2 μg kg^−1^ IV (Fentadon 50 μg mL^−1^, Dechra Veterinary Products S.L.U., Barcelona, Spain). If the anaesthetic plane became superficial (indicated by positive blink reflex or increased mandibular tone), propofol boluses were administered (effect dose) or the concentration of isoflurane was increased.

At the end of the procedure, using a visual analogue scale (VAS), the surgeon assessed the overall quality of the arthroscopy image and the satisfaction of the arthroscopic procedure. The VAS was represented as a line from 0 to 100 mm, where 0 mm was no visibility and 100 mm was perfect visibility. In the case of the degree of satisfaction on the part of the orthopaedic surgeon with the arthroscopy, 0 mm represented that the procedure had presented many complications, and he was not satisfied and 100 mm represented that the procedure had passed without complications. The duration of the procedure, the duration of the anaesthesia and the total irrigation fluids used will be recorded. The remaining fluid in the arthroscopic collection bag and the aspirator will be counted, and a sample will be taken in ethylenediaminetetraacetic acid (EDTA) tube and store at −80 °C, for subsequent determination of haemoglobin concentration by the cyano-haemoglobin technique [37].

**Figure 1:**
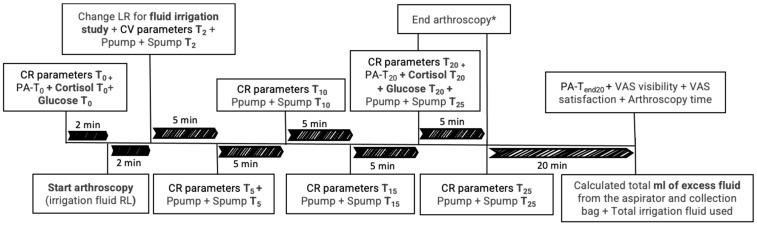
Chronological timeline of variables collected throughout the study, including cardiorespiratory (CR) parameters, pressure and speed of the pumping system (Ppump and Spump), cortisol levels, glucose levels, plasma adrenaline (PA), excess arthroscopy irrigation fluid, total irrigation fluids used, arthroscopy image quality, satisfaction of the arthroscopic procedure (VAS visibility and VAS satisfaction), and duration of the arthroscopy. T_0_ (2 min before starting the arthroscopy), T_2_ (within 2 min of waiting after the entrance of the optic), T_5_ (after 5 min of starting with the study irrigation fluid), every 5 min until the end of the procedure (T_10_, T_15_, T_20_ and T_25_), and T_end20_ (20 min after finishing the arthroscopy). Lactate Ringer (LR). * The arthroscopy was completed at 20 min in some dogs and at 25 or 30 min in others, depending on the surgeon’s discretion.

Three blood extractions from the jugular catheter were performed. For the first two extractions, at T_0_ and T_20_, glucose was measured immediately using a glucometer (iPetPRO Ultimed, Inc., Excelsior, MN, USA). Additionally, 2 mL were placed in a serum tube with gel, and 3 mL in an EDTA tube. The last extraction, at T_end20_ (20 min after finishing the arthroscopy), was 3 mL in an EDTA tube. The three EDTA tubes were centrifuged immediately after extraction for 5 min at 3500 rpm and the plasma was stored in an Eppendorf (PA-T_0_, PA-T_20_ and PA-T_end20_) at −80 °C for subsequent determination of concentration of plasma adrenaline (PA). The gel tubes were centrifuged one hour later for 5 min at 3500 rpm and the serum was stored in an Eppendorf at −80 °C to measure cortisol (cortisol T_0_ and cortisol T_20_) (Figure 1).

The short form of the Glasgow composite measure pain score (CMPS-SF) was evaluated before surgery (T_CMPS-basal_) and at 2, 4 and 24 h (T_CMPS-2_, T_CMPS-4,_ T_CMPS-24_) after extubation, were performed. During hospitalization, the use of trazodone, methadone, and non-steroidal anti-inflammatory drugs (NSAIDs) was also recorded.

### 2.4. Laboratory Analysis Blood Samples

#### 2.4.1. Clotting Times

Partial thromboplastin time (PTT) and activated partial thromboplastin time (aPTT) values were measured within 30 min after extraction (IDEXX Coag DXTM, IDEXX Laboratories, Inc., Westbrook, ME, USA).

#### 2.4.2. Haemoglobin

The quantitative determination of haemoglobin was performed using the cyanohaemoglobin method (Drabkins Reagent 100 mL, Spinreact, S.A./S.A.U., Barcelona, Spain) described by Kampen and Zijlstra (1961) [37]. The vacutainer containing the 4 mL sample of the supernatant arthroscopic irrigation fluid, previously stored at −80 °C, was thawed at room temperature for a couple of hours. It was then centrifuged for 10 min at 3500 rpm (Nahita Blue 2650-D+, Auxilab S.L., Navarra, Spain). After centrifugation, the supernatant was aspirated and deposited into separate tubes for each sample in duplicate, leaving the sediment or debris at the bottom of the original tube. Following this, Drabkin’s reagent was added to all tubes. The tubes were mixed well and allowed to rest for 3 min at room temperature (15–25 °C). Subsequently, absorbance was measured at a wavelength of 540 nm using a spectrophotometer (VarioskanTM LUX multimode microplate reader, Thermo Fisher Scientific Inc.,Waltham, MA, USA). The haemoglobin value in g dl^−1^ was then multiplied by the total volume of the supernatant arthroscopic irrigation fluid to determine the total haemoglobin in grams.

#### 2.4.3. Cortisol

The serum samples were stored frozen at −80 °C until all collections were complete and were then analysed at an external laboratory (Cedivet Valencia, S.L., Valencia, Spain). After being thawed at room temperature for a few hours, the samples were processed using an in vitro chemiluminescence immunoassay kit for the quantitative determination of cortisol, employing the fully automated MAGLUMI^®^ 800 analyser (Snibe Co., Ltd., Shenzhen, China), the results were expressed in μg dL^−1^.

#### 2.4.4. Adrenaline

The adrenaline concentration was quantified using the competitive Elisa test (Adrenaline Research Elisa, ref. DEE5100R, Demeditec Diagnostics GmbH, Kiel, Germany). Once all samples were collected, they were thawed for 4 h at room temperature, followed by centrifugation at 3500 rpm for 10 min. The resulting supernatant was used to measure adrenaline concentration according to the protocol provided by the ELISA kit. The final concentration of the samples was adjusted by multiplying by a correction factor. Results were expressed in pg mL^−1^.

### 2.5. Statistical Analysis

The sample size was calculated based on a 20% increase in the HR of group A compared to group C. To achieve a power of 90% and a significance of *p* < 0.05, 10 diagnostic knee arthroscopies per group were required. This calculation was based on the data from a pilot study by Roman and Serra (2021) [38]. Considering the possibility of excluding some knee arthroscopies from the clinical trial, the final sample size was adjusted to include an additional 10% of the total number of knee arthroscopies needed.

The statistical analysis was conducted using R language (version 4.4.2). Continuous variables, such as HR, SAP, MAP, DAP, and RR, were summarised using medians and interquartile ranges. We reported frequencies for ordinal variables.

Boxplots were created to visualise data distributions and temporal trends, stratified by group and time to highlight central tendencies and variability. Generalised linear models (GLMs) were used to assess the effects of group, time, and their interaction on continuous outcomes. These models treated time as a categorical factor to account for its discrete nature and included interaction terms to examine differences in trends between groups. Model outputs provided coefficients, standard errors, and *p*-values, facilitating the interpretation of group-specific effects over time. Mann–Whitney U tests were performed for non-parametric outcomes to compare distributions between groups, addressing the non-normality of variables such as VAS scores. We evaluated the relationships between total haemoglobin levels and continuous variables using Spearman’s rank correlation coefficients, which are robust to deviations from normality and can identify monotonic relationships. We compared trends for both groups in analysing time-based variables such as hemodynamic advanced parameters. These comparisons were visualised using boxplots, and GLMs were applied to test for significant group-by-time interactions. Statistical significance was set at *p* < 0.05.

## 3. Results

### 3.1. Clinical Cases

Twenty-two knee arthroscopies were enrolled in the study. Two were excluded, one because it was not successful to place an arterial catheter and the other one because the adrenaline samples were not centrifuged and frozen within the established times. In total twenty knee arthroscopies (10 in group C and 10 in group A) from 18 client-owner dogs, eleven male and seven female, were analysed. Bilateral procedures were performed in two dogs, and these were performed in two isolated procedures with 4 months between them. Analysis of dog demographics found no significant difference regarding age, sex, weight, body condition score (BCS) or ASA physical status between groups (ASA II status reflects increased anaesthetic risk due to obesity and/or advanced age rather than specific systemic disease). The demographic details can be found in Table 1. The dogs belonged to various breeds. The most common breeds were mixed-breed, accounting for 30% of the population, while pure breeds made up the remaining 70%, including American Bullies (10%), Labrador Retrievers (10%), White Swiss Shepherds (10%), Cane Corso (5%), Golden Retriever (5%), German Shepherd (5%), Pit Bull Terrier (5%), American Staffordshire terrier (5%), Spanish water shepherd (5%), Beagle (5%), and Dalmatian (5%).

Three dogs were in treatment with drugs that can alter platelet aggregation (NSAIDs), two from group C and one from group A. Seven dogs (35%) in Group A and six dogs (30%) in Group C received mechanical ventilation. During the regional nerve block, three complications were noted in group A: in two cases, there was an unclear visualization of the saphenous nerve, and in one case, blood was observed during aspiration prior to the injection of local anaesthetic for the saphenous nerve block. During arthroscopy, nine dogs in Group A exhibited nociception; in four of them, the stimulus occurred when incising the cranial-lateral skin region of the knee, and in another four, during manipulation of the caudal joint capsule of the knee, with one dog showing nociception at both previously mentioned points. In Group C, five dogs exhibited nociception: one during incision of the cranial-lateral skin region of the knee, three during manipulation of the caudal joint capsule, and dog showed nociception unrelated to the aforementioned points. Of the patients with nociception, only two in Group C and four in Group A required rescue analgesia with fentanyl. Additionally, two dogs in group A and one dog in group C required propofol to deepen the anaesthetic plane during the procedure. One dog in group A experienced hypotension (MAP < 60 mmHg), which was resolved with a single IV dose of ephedrine at 50 μg kg^−1^ (Hidrocloruro de efedrina Kabi 30 mg mL^−1^, Fresenius Kabi España S.A.U., Barcelona, Spain).

### 3.2. Cardiorespiratory Parameters

**Figure 2:**
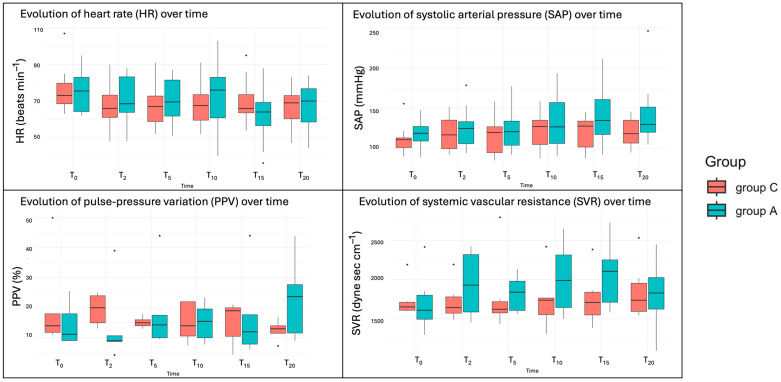
Boxplots of the cardiovascular variables over time. T_0_ (2 min before starting the arthroscopy), T_2_ (within 2 min of waiting after the entrance of the optic), T_5_ (after 5 min of starting with the study irrigation fluid), and every 5 min until the end of the procedure (T_10_, T_15_ and T_20_).

The analysis of cardiorespiratory variables including HR, RR, SAP, MAP, and DAP revealed no statistically significant differences between the two treatment groups across the measured time points, nor for the same group over time (*p* > 0.05). There was a tendency towards higher values in Group A for SAP, HR, and RR but these differences were not statistically significant (Figure 2).

The advanced haemodynamic variables, including CO, SV, SVR, dP/dtmax, SPV, PPV, and CCE, were measured with the Mostcare^®^ monitor, but data were collected from only six dogs in each group. These variables did not show significant changes attributable to the fluid irrigation protocol across time points, nor for the same group over time (all *p* > 0.05). However, there was a tendency toward higher values and greater variability in group A for CO and SVR, though these differences were not statistically significant. Additionally, group A showed a tendency for greater variability in PPV values at 20 min (Figure 2).

### 3.3. Arthroscopy Parameters

**Figure 3:**
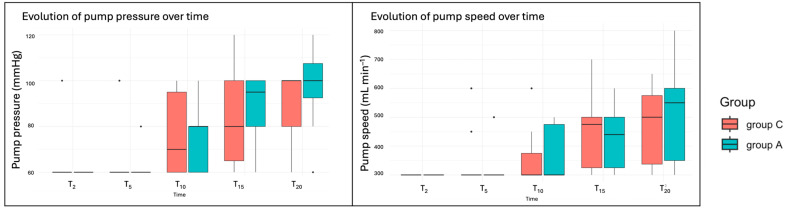
Boxplots of the irrigation pump variables. T_2_ (within 2 min of waiting after the entrance of the optic), T_5_ (after 5 min of starting with the study irrigation fluid), and every 5 min until the end of the procedure (T_10_, T_15_, and T_20_).

The amount of irrigation fluid used was 460 mL [200–600] in group C and 190 mL [90–1020] in group A, with no significant differences between them (*p* = 0.34). However, there was high variability in the adrenaline group. Similarly, the operative time was comparable between groups. The median arthroscopy duration was 23 m in [21–27] for Group C and 23 min [21–32] for Group A. Since the duration of the arthroscopy varied between dogs, 21 min was the minimum, with some procedures lasting up to 30 min. However, only cases up to T_20_ were analysed, as only a few cases exceeded this duration. In three dogs from group C and one dog from group A, minor complications occurred during the procedure, including loss of the drainage port, issues with the irrigation pump (presence of air bubbles), and the need to change the size of the optical. Significant differences between times were observed at T_15_ and T_20_ in terms of increased pump pressure or speed during the procedure (*p* = 0.004, *p* = 0.0006 and *p* = 0.002, *p* = 0.0005, respectively), but not between groups (*p* = 0.55) (Figure 3). 

### 3.4. Image Visualisation and Satisfaction of the Procedure

No statistically significant difference in surgeon-rated clarity of the visual field, based on VAS scores was found for all arthroscopic knee procedures, the median was 97 [52–100] mm for group C and 89.5 [67–100] mm for group A. The satisfaction of the arthroscopy procedure was similar between groups 88.5 [3–100] mm for group C and 94.5 [26–100] mm for group A.

### 3.5. Glasgow Scale

**Figure 4:**
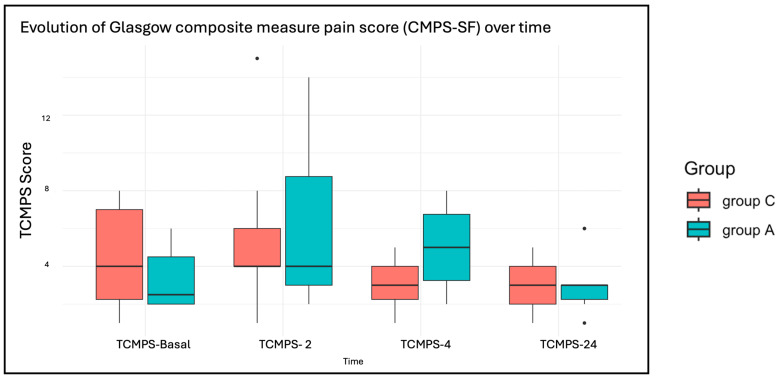
Boxplots of the evaluation of short form of the Glasgow composite measure pain score (CMPS-SF) at multiple time points, before surgery (T_CMPS-basal_) and at 2, 4 and 24 h (T_CMPS-2_, T_CMPS-4,_ T_CMPS-24_) after extubation.

The assessment of postoperative pain evaluated using the Glasgow CMPS-SF at multiple time points (T_CMPS-Basal_, T_CMPS-2_, T_CMPS-4_, T_CMPS-24_) showed a significant reduction in pain scores over time in both groups (*p* = 0.036). However, no significant differences in pain reduction were detected between the two treatments (*p* = 0.21). Although Group A exhibited a broader range of values, with a tendency toward higher results on the scale (Figure 4). All dogs received methadone during the first 12 h postoperatively, followed by buprenorphine as needed. Different NSAIDs were administered among the dogs: seven in Group A and seven in Group C received meloxicam, while robenacoxib was administered to one dog in Group A and two dogs in Group C. One dog in each group received firocoxib, and one dog in Group A was treated with carprofen. Additionally, six dogs in Group C and one dog in Group A received trazodone as an anxiolytic.

### 3.6. Laboratory Results

**Figure 5:**
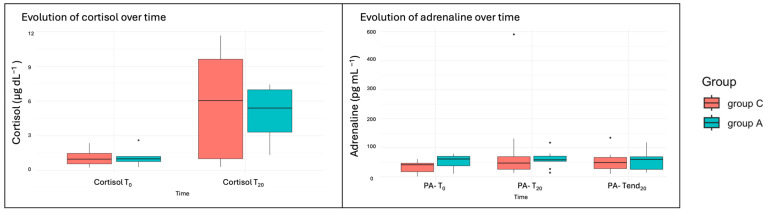
Boxplots of the cortisol and plasma adrenaline (PA) variables. T_0_ (2 min before starting the arthroscopy), T_20_ (after 20 min of starting with the study irrigation fluid), and T_end20_ (20 min after finishing the arthroscopy).

Clotting times were within the normal range for all dogs, except for one dog who exhibited an increased aPTT. This dog, which was not treated with NSAIDs, underwent two arthroscopic procedures and was included in both the adrenaline and control groups. Glucose levels did not differ significantly between groups (*p* = 0.26), although a slight increase was observed after 20 min of starting with the study irrigation fluid in both groups (*p* = 0.49). Similarly, cortisol levels increased during de-arthroscopy in both groups (*p* = 0.007) but showed no significant differences between treatment groups (*p* = 0.97). Adrenaline levels showed a significant increase at PA-T_20_ in both groups (*p* = 0.041) (Table 2). Although no significant differences were observed between groups (*p* = 0.54), outliers were present in both groups but were more prominent in Group A (Figure 5).

The calculated intra-articular bleeding during the arthroscopy with the haemoglobin concentration of the supernatant fluid was similar between groups (*p* = 0.86) and no correlations between haemoglobin and VAS scale were statistically significant (*p* > 0.05). The strength of the relationships ranged from weak to moderate. However, VIS (ρ = −0.287) was still not statistically significant. Clinically, no consistent relationship could be established between the total haemoglobin levels and the subjective assessments of visibility and surgeon satisfaction in this analysis.

## 4. Discussion

The addition of adrenaline to intra-articular lavage fluid at a concentration of 0.33 mg L^−1^ did not affect cardiorespiratory parameters during diagnostic arthroscopic procedures, making this a safe approach for clinical use. This finding is consistent with data from human medicine, which indicate no adverse cardiovascular reactions—specifically in HR, SAP, MAP, and DAP during shoulder arthroscopies [17,32,33]. To the authors’ knowledge, no published studies in human medicine have employed advanced hemodynamic monitoring to assess the effects of adding adrenaline to arthroscopic irrigation fluid. In our study, we used the PRAM method (MostCare^®^) for every case whenever it was available; however, on days when it was occupied with critically ill dogs (ASA score > III) in another operating room, we could not include additional study dogs. Because priority was given to those high-risk cases, we are unable to reach our planned sample size. This study is also the first to evaluate the use of adrenaline as a haemostatic agent in arthroscopy in dogs, making it difficult to compare our findings with other veterinary research.

We delayed the introduction of the study irrigation fluid by 2 min of unsupplemented LR to obtain unperturbed baseline cardiorespiratory parameters and verify that both groups started from identical physiological conditions. Portal insertion and joint distension provoke transient sympathetic activation—raising heart rate and arterial pressure—which could confound later comparisons if the supplemented fluid were applied immediately. By waiting, we allowed these effects to stabilise; any significant differences at T_2_ would have revealed unequal baselines, preventing valid attribution of changes at T_5_ and beyond to adrenaline versus the control group. This step was therefore critical to ensure that observed effects truly reflect the intervention. In addition, the initial minutes of arthroscopy allowed exploration of the joint and assessment of inflammatory changes in the synovial membrane, which could be misleading if adrenaline-induced vasoconstriction were already present.

The effects of adrenaline on the cardiorespiratory system are dose-dependent. At low doses (0.01 mg kg^−1^ IV), β_1_ and β_2_-adrenergic agonist effects predominate, resulting in increased CO, decreased DAP, and peripheral vascular resistance. At higher doses (0.1 mg kg^−1^ IV), α_1_-adrenergic effects become more pronounced, leading to a marked rise in SVR [39]. The recommended concentration of adrenaline for arthroscopic fluid in human medicine, and the one used in our study, was 0.33 mg L^−1^ [18]. Accordingly, advanced haemodynamic monitoring, such as the PRAM method (MostCare^®^) [40,41], was employed to detect any subtle changes in CO and SVR. The progressive increase in SAP, CO, and SV throughout the study could be attributed to various factors, although the authors do not have a clear hypothesis. One possible explanation is the increase in the infusion pressure of the adrenaline irrigation pump, which corresponds to the rise in values observed in the adrenaline group starting at 15 min into the study. However, if this were the primary cause or the result of a cumulative effect over the course of the procedure, a significant increase in adrenaline concentrations would have been expected, which was not the case. It cannot be ruled out that the alteration of cardiovascular parameters may be due to the fact that in the adrenaline group, some blocks did not provide complete analgesia. Although only two dogs in Group A presented technical difficulties during the ultrasound-guided saphenous nerve block, and the remaining blocks were performed successfully with confirmed deposition of local anaesthetic, some animals exhibited transient nociceptive responses during specific stages of the procedure, particularly during manipulation of the craniolateral skin and the caudal joint capsule. This can be attributed to the fact that, as described in the literature, certain sensory branches may remain uncovered with the standard proximal saphenous block approach. Specifically, the medial articular branch of the saphenous nerve [42] and obturator nerve branches [43] might not be consistently desensitised. However, the lateral femoral cutaneous nerve (LFCN) is not affected by this block and requires a separate injection to be desensitised [42]. While excluding these patients from the analysis was considered, we believe these responses reflect real-world limitations of commonly used locoregional anaesthesia protocols in clinical settings [44,45], rather than a failure in technique. Importantly, these findings did not result in statistically significant differences between groups, and the overall analgesic management was deemed clinically effective. Therefore, while we do not consider it necessary to exclude these patients, future studies could explore broader locoregional techniques (e.g., lumbar plexus compartment block and LFCN) to assess whether more extensive desensitisation influences intraoperative nociceptive responses or hemodynamic variability.

The PPV% values in this study should be interpreted cautiously, as mechanical ventilation was only provided to obese dogs, those experiencing apnoea after induction, or dogs with moderate hypercapnia. Mechanical ventilation increases pressure within the thoracic cavity, potentially compressing large blood vessels, and reducing venous return and CO. These hemodynamic changes can lead to elevated PPV values. Our study evaluated PPV in all dogs, regardless of whether they were on mechanical ventilation. The authors observed a progressive increase in PPV throughout the arthroscopic procedure in the adrenaline group, but no clear explanation for this finding is evident. Interestingly, the control group had the same number of dogs under mechanical ventilation as the adrenaline group. Conversely, a study evaluating PPV in dogs undergoing orthopaedic procedures under mechanical ventilation observed that up to 62.5% of normotensive dogs under inhalation anaesthesia responded to fluids with PPV values greater than 15% [40]. Nevertheless, the authors did not treat dogs who were on mechanical ventilation and exhibited a PPV increase above 15%, as they were normovolemic, normotensive, and without tachycardia. The dog with hypotension was treated with a vasopressor.

Although there have been isolated case reports in human medicine describing complications such as severe hypertension, tachycardia, paroxysmal supraventricular tachycardia complicated by ventricular tachycardia, fibrillation, pulmonary oedema, and cardiac arrest, these complications are rare [34,35,46,47]. This does not imply that the technique is without risk; therefore, we recommend considering adrenaline supplementation in the irrigation fluid as a potential cause if acute hypertension, tachycardia, or hypoxaemia are observed during the arthroscopic procedure. Chierichini et al. [20] found that during shoulder arthroscopies, adrenaline was associated with a higher incidence of hypotensive and bradycardic events compared to noradrenaline. This suggests that noradrenaline may be a safer alternative for patients with cardiovascular risk. Several hypotheses have been proposed to explain the mechanism by which adrenaline causes adverse cardiorespiratory effects. One significant hypothesis is the inadequate mixing of adrenaline in the arthroscopic irrigation fluid, leading to a high adrenaline concentration over a short period. Another possibility is hypersensitivity to alpha-adrenergic agonists due to systemic absorption through an intraosseous route, even at minimal concentrations [34]. Our study suggests that intraosseous absorption of adrenaline either does not occur or does not reach plasma levels sufficient to alter significantly cardiorespiratory parameters during diagnostic knee arthroscopies. This may be because the procedures were diagnostic in nature, with no manipulation of the cartilage, subchondral bone, or synovial membrane. We hypothesise that there might be a greater risk of adverse cardiorespiratory effects in dogs undergoing therapeutic arthroscopies, which involve more extensive tissue handling. The prolonged exposure to adrenaline from the arthroscopic irrigation fluid during therapeutic procedures could increase the likelihood of such effects, potentially altering the safety profile compared to shorter diagnostic arthroscopies. Furthermore, in our study, the subjective visibility scores were already very high in both groups, suggesting that the technical protocol used provided excellent visualization regardless of adrenaline administration. This limited margin for improvement may have masked any potential benefit of adrenaline. Considering this, along with the potential risks of dosing or handling errors, its routine use in diagnostic stifle arthroscopies may not be justified. Future research should focus on therapeutic procedures to better understand the potential risks and mechanisms involved, particularly studies that evaluate advanced hemodynamic parameters with an adequately sized sample, to provide more comprehensive insights.

The use of adrenaline as a haemostatic agent has been described in veterinary medicine for intracameral administration in dogs undergoing phacoemulsification [29], via subserosal injection for acute gastric bleeding [48], and in bilateral maxillary nerve block in dogs undergoing staphylectomy [30]. These techniques are reported as safe, with no observed haemodynamic complications. Until now, the use of adrenaline as a haemostatic agent added to arthroscopic irrigation fluid in dogs has not been described, although its intra-articular use combined with local anaesthetics for analgesia has been reported [49]. Contrary to these previous studies where adrenaline reduced bleeding, our study did not observe any benefits. Haemoglobin levels in the fluid and the degree of visibility were similar between groups. This discrepancy could be due to differences in administration techniques. In our case, the arthroscopic fluid containing adrenaline continuously irrigated the joint, so it was in contact with the tissue for less time, unlike the previously mentioned studies where a single local dose of diluted adrenaline was administered, and this was absorbed by a longer contact time with the tissue. This continuous irrigation could make it difficult for clots to form and cessation of bleeding, despite local vasoconstriction. In human medicine, a reduction in bleeding was observed in patients undergoing arthroscopy with adrenaline-containing irrigation fluid compared to those using only physiologic saline solution [17,32,33]. We hypothesise that the difference in our results could be attributed to the nature of the procedure, as previously explained. Both groups in our study showed high visibility and procedural satisfaction, indicating minimal bleeding and adequate procedure performance. In contrast, therapeutic arthroscopies, which involve more extensive bleeding due to synovectomy or cartilage debridement, might benefit from adrenaline use, similar to findings in human studies. Another factor to consider is the role of haemostatic mechanisms, with possible differences between species contributing to the observed discrepancies. By examining the use of adrenaline in different contexts and understanding the variability in techniques and biological responses, we can better tailor its application to maximise benefits while ensuring safety across species.

The CMPS-SF results did not indicate better postoperative comfort for dogs in the adrenaline group. This is likely because the dogs underwent orthopaedic surgeries following arthroscopy. Therefore, postoperative pain associated solely with the arthroscopy could not be evaluated. Additionally, the trend observed in both groups showed a progressive decrease in pain during the hours following the procedure, suggesting that the analgesic management was appropriate. This likely made it difficult to detect subtle differences between groups. Although the control group tended to show lower scores on the scale, postoperative analgesic requirements were similar between groups. It is noteworthy that six dogs in the control group received trazodone as an anxiolytic. This may have reduced stress levels and contributed to greater comfort during hospitalization, which the authors hypothesise could explain the observed tendency for lower Glasgow CMPS scores in the control group.

In the study, glucose, cortisol, and adrenaline levels were similar between groups. This finding may be attributed to the minimal intraosseous absorption of adrenaline during the procedure, as well as to the fact that the concentration of adrenaline used was not sufficiently elevated to induce significant changes in cortisol and glucose levels in the adrenaline group. Elevated catecholamines in the blood typically lead to increased cortisol and glucose [50,51,52], but local administration of adrenaline in small doses and for a brief duration may not have reached systemic circulation levels sufficient to induce a significant endocrine response. Goel et al. (2016) confirmed that local adrenaline administration might not cause changes in plasma glucose levels if adrenaline is not adequately absorbed into the bloodstream [53]. The levels of glucose, cortisol and adrenaline increased at the 20 min of start arthroscopy in both groups compared to baseline values. This increase may be attributed to nociceptive stimuli [54,55,56] occurring during the arthroscopic procedure, as six dogs required intraoperative rescue analgesia during the arthroscopy. While the regional nerve block was effective, it did not provide complete analgesia at specific moments during the arthroscopy, such as during the insertion of the trocars into the joint or the manipulation of the caudal aspect of the capsule. Additionally, although no significant changes in cardiovascular values were observed in the other 14 dogs to suggest marked nociceptive responses, this slight trend indicates that some level of nociception may still have been present [57]. Furthermore, the manipulation of adrenaline samples after extraction from dogs could introduce a degree of metabolic degradation, resulting in values that do not accurately reflect the concurrent increases in cortisol and glucose. This potential alteration in adrenaline levels emphasises the importance of careful handling and timely analysis of hormonal samples to ensure reliable interpretations of the physiological stress response during surgical interventions [58,59].

The study presented several limitations. First, the exposure to adrenaline lasted a maximum of 31 min in the longest case, although the data were analysed only up to 20 min and was conducted during diagnostic arthroscopies. Consequently, with longer contact time and its use in therapeutic arthroscopies where cartilage and subchondral bone damage occur, the results may differ from those obtained in this study [17]. In addition, the necessary sample size to evaluate advanced cardiovascular parameters and image visualization was not achieved, as monitoring equipment was often needed for more critically ill dogs, limiting its availability for this study. Additionally, the VAS used to evaluate image quality and surgical satisfaction is subjective and could introduce bias. The surgeon’s experience and expectations may influence the scores [32,60]. Furthermore, this study only investigated the effects of a single adrenaline concentration (0.33 mg L^−1^). Different concentrations may have varying effects on cardiovascular parameters and haemostasis [21]. The results for adrenaline should be interpreted with caution, as external variables, such as handling and storage conditions, may influence the outcomes. Adrenaline is highly susceptible to degradation if not maintained at low temperatures during processing. Therefore, it is essential that samples be centrifuged within a defined time frame and promptly frozen to preserve their integrity [61,62]. Any deviation from these protocols could result in unreliable adrenaline levels, potentially affecting the accuracy of comparisons with other markers, such as cortisol and glucose. Therefore, including various types of arthroscopic procedures to evaluate the haemostatic and cardiovascular effects of adrenaline in more invasive therapeutic arthroscopies could yield different results.

## 5. Conclusions

The addition of adrenaline to intra-articular lavage fluid at a concentration of 0.33 mg L^−1^ during diagnostic stifle arthroscopy in healthy dogs appears to be a safe approach, as it did not significantly affect cardiorespiratory parameters. However, adrenaline did not improve arthroscopic visualization; therefore, its use at this concentration does not appear to provide a clinical benefit in diagnostic stifle arthroscopies. Further studies are needed to evaluate its potential role in therapeutic arthroscopies involving more bleeding and longer surgical times.

## Figures and Tables

**Table 1:** Demographic characteristics of the studied population and time of anaesthesia. Data are presented as frequency tables or median [range].

Variable	Group C (n = 10)	Group A (n = 10)	Overall (n = 20)	*p*-Value
Sex				1.00
Male	6 (60.0%)	7 (70.0%)	13 (65.0%)	
Female	4 (40.0%)	3 (30.0%)	7 (35.0%)	
Age (years)	3.5 [1.2–11.0]	7 [1.8–12.0]	5.5 [1.2, 12]	0.24
Weight (kg)	34.5 [16.6–49]	29.5 [9–40]	32.8 [16.6–49]	0.57
Body condition score (BCS)				0.66
Ideal (4–5/9)	5 (50.0%)	6 (60.0%)	11 (55.0%)	
Overweight (6–7/9)	3 (30.0%)	2 (20.0%)	5 (25.0%)	
Obese (8–9/9)	2 (20.0%)	2 (20.0%)	4 (20.0%)	
ASA physical status				1.00
ASA I	8 (80.0%)	7 (70.0%)	15 (75.0%)	
ASA II	2 (20.0%)	3 (30.0%)	5 (25.0%)	
Time of anaesthesia (min)	275 [235–300]	235 [160–305]	270 [160–305]	0.10

**Table 2:** Descriptive analysis of glucose (mg dL^−1^), cortisol (µg dL^−1^) and plasma adrenaline (PA) (pg mL ^−1^) variables (mean and median) at T_0_ (2 min before starting the arthroscopy), T_20_ (after 20 min of starting with the study irrigation fluid), and T_end20_ (20 min after finishing the arthroscopy).

Variable	Group	Mean	SD	Median	Range
Glucose T_0_	Group C	126.50	24.78	122.50	[98–166]
Glucose T_20_	Group C	138.00	16.11	132.00	[122–161]
Glucose T_0_	Group A	145.67	34.33	131.50	[107–195]
GlucoseT_20_	Group A	163.83	34.11	150.50	[132–215]
Cortisol T_0_	Group C	1.10	0.78	0.98	[0.25–2.37]
Cortisol T_20_	Group C	5.61	4.81	6.04	[0.32–11.66]
Cortisol T_0_	Group A	1.15	0.79	1.00	[0.29–2.61]
Cortisol T_20_	Group A	4.94	2.48	5.39	[1.33–7.43]
Adrenaline T_0_	Group C	33.87	20.35	41.17	[1.04–60.84]
Adrenaline T_20_	Group C	94.06	143.50	47.05	[12.53–490.71]
Adrenaline T_end20_	Group C	52.43	36.06	48.80	[9.92–134.38]
Adrenaline T_0_	Group A	51.61	24.35	61.08	[10.41–79.64]
Adrenaline T_20_	Group A	60.21	28.28	58.28	[14–117.38]
Adrenaline T_end20_	Group A	54.11	32.50	59.82	[14.17–118.4]

## Data Availability

The datasets used and/or analysed during the current study are available from the corresponding author upon reasonable request.
